# Adult Patients’ Experiences of Using a Patient Portal With a Focus on Perceived Benefits and Difficulties, and Perceptions on Privacy and Security: Qualitative Descriptive Study

**DOI:** 10.2196/46044

**Published:** 2023-07-25

**Authors:** Elisa H Son, Eun-Shim Nahm

**Affiliations:** 1 Translational Biobehavioral and Health Disparities Branch National Institutes of Health Clinical Center Bethesda, MD United States; 2 Organizational Systems and Adult Health University of Maryland School of Nursing Baltimore, MD United States

**Keywords:** patient portal, patient engagement, user review, content analysis, qualitative, perception, privacy, security

## Abstract

**Background:**

Patient portals can facilitate patient engagement in care management. Driven by national efforts over the past decade, patient portals are being implemented by hospitals and clinics nationwide. Continuous evaluation of patient portals and reflection of feedback from end users across care settings are needed to make patient portals more user-centered after the implementation.

**Objective:**

The aim of this study was to investigate the lived experience of using a patient portal in adult patients recruited from a variety of care settings, focusing on their perceived benefits and difficulties of using the patient portal, and trust and concerns about privacy and security.

**Methods:**

This qualitative descriptive study was part of a cross-sectional digital survey research to examine the comprehensive experience of using a patient portal in adult patients recruited from 20 care settings from hospitals and clinics of a large integrated health care system in the mid-Atlantic area of the United States. Those who had used a patient portal offered by the health care system in the past 12 months were eligible to participate in the survey. Data collected from 734 patients were subjected to descriptive statistics and content analysis.

**Results:**

The majority of the participants were female and non-Hispanic White with a mean age of 53.1 (SD 15.34) years. Content analysis of 1589 qualitative comments identified 22 themes across 4 topics: beneficial aspects (6 themes) and difficulties (7 themes) in using the patient portal; trust (5 themes) and concerns (4 themes) about privacy and security of the patient portal. Most of the participants perceived the patient portal functions as beneficial for communicating with health care teams and monitoring health status and care activities. At the same time, about a quarter of them shared difficulties they experienced while using those functions, including not getting eMessage responses timely and difficulty finding information in the portal. Protected log-in process and trust in health care providers were the most mentioned reasons for trusting privacy and security of the patient portal. The most mentioned reason for concerns about privacy and security was the risk of data breaches such as hacking attacks and identity theft.

**Conclusions:**

This study provides an empirical understanding of the lived experience of using a patient portal in adult patient users across care settings with a focus on the beneficial aspects and difficulties in using the patient portal, and trust and concerns about privacy and security. Our study findings can serve as a valuable reference for health care institutions and software companies to implement more user-centered, secure, and private patient portals. Future studies may consider targeting other patient portal programs and patients with infrequent or nonuse of patient portals.

## Introduction

### Background

Patient engagement in care management, such as informed choices and shared decision-making, is emphasized in current health service delivery [1,2]. The widespread use of health information technologies (HITs) has enabled patients to be more actively involved in their care activities [3]. A patient portal is a type of HIT linked to electronic health records (EHRs) that allows patients to view their medical records, communicate with care providers, and perform other care-related tasks [4]. Driven by national efforts over the past decade, patient portals are being widely implemented by hospitals and clinics across the nation [5].

A large body of patient portal research has focused on investigating the effects of using patient portals or factors that may influence patient portal adoption. Researchers have demonstrated the positive effects of using patient portals on patient engagement in care activities (eg, appointment adherence and medication management) and clinical health outcomes (eg, blood pressure and blood glucose control) [6,7]. Regarding factors associated with patient portal adoption, perceived usability has been discussed as a main factor [8-10]. Common usability issues included problems with log-in or access and difficulties in understanding information or navigating functions in patient portals [8,9]. Another notable factor associated with patient portal adoption was concerns about privacy and security risks [11]. Primary concerns were disclosure of personal health information to others outside of one’s permission or unauthorized use of the information by third parties [11]. While usability issues and privacy concerns were the main barriers, health care providers’ recommendations and training support were facilitating factors of patient portal adoption [12,13].

According to a national survey (N=3865) conducted in 2020, about 60% of people were offered digital access to medical records, and about 64% of them accessed their medical records in the past 12 months [14]. As the percentage of patients using patient portals increases, the perception and experience of using patient portals may vary among the users from diverse demographic and clinical backgrounds [8,15]. Continuous evaluation of patient portals and reflection of feedback from end users across care settings are needed to make patient portals more user-centered after the implementation. Existing studies examining patients' experiences of using patient portals often included small samples recruited from limited clinical or research environments [2,8,16]. There still is a lack of empirical understanding of the lived experience of using patient portals in larger samples recruited across care settings.

### Objective

To fill the current gap in patient portal research, we conducted an anonymous digital survey of adult patients recruited from a variety of care settings to examine their comprehensive experiences of using a patient portal [17]. As part of the survey, the participants submitted qualitative comments on their perceived benefits and difficulties of using the patient portal and perceptions on privacy and security. The aim of this study was to investigate the lived experience of using the patient portal in adult patients recruited across care settings, focusing on their perceived benefits and difficulties of using the patient portal, and trust and concerns about privacy and security.

## Methods

### Study Design and Participants

This qualitative descriptive study was part of a cross-sectional digital survey research to investigate the comprehensive experience of using a patient portal in adult patients who had accessed the portal in the past 12 months [17]. The participants were recruited from 20 care settings in hospitals and clinics of a large integrated health care system in the mid-Atlantic region of the United States; the selected care settings represented various geographical locations (urban and rural), treatment areas (primary and special), and patient portal activation densities (large and low). Inpatients or outpatients who were 18 years or older, had an active patient portal account offered by the health care system (MyChart by Epic Systems Corporation), and had used the portal at least twice in the past 12 months prior to the survey were eligible for participation. A 1-time anonymous survey was administered from August 19 to September 20, 2019. An eMessage that includes a hyperlink to the web-based survey with a brief invitation was sent to 9949 patients who had visited the selected care settings a week prior to the start of the survey. A total of 743 patients participated in the survey, and data from 734 patients who responded to at least one open-ended question were included in the current analysis.

### Selected Data and Measures

Demographic and descriptive variables included age, sex, race or ethnicity, marital status, education, monthly income, employment, presence of chronic disease, and internet usage hours per week. eHealth literacy was measured using the eHealth Literacy Scale, an 8-item 5-point Likert scale (1=strongly disagree; 5=strongly agree) [18]. The eHealth Literacy Scale is internally consistent and valid [19,20], and it had a Cronbach α of .93 in this study. The perceived usability of the patient portal was measured using a modified Perceived Health Web Site Usability Questionnaire, a six-item 7-point Likert scale (1=strongly disagree or very unsatisfied; 7=strongly agree or very satisfied) [21]. Cronbach α of Perceived Health Web Site Usability Questionnaire was .92 in this study. Concerns about privacy and security of the patient portal were measured using a single item on a 7-point Likert scale (1=not at all worried; 7=very worried) adopted from the National Consumer Survey on HIT conducted for California HealthCare Foundation [22]. The frequency of patient portal use in the past 12 months was measured using a single ordinal item (1 to 9 times, about monthly, more than monthly).

The participants were further asked open-ended questions about beneficial aspects of the patient portal, specific difficulties that they experienced when using the patient portal, and their trust in privacy and security of the patient portal. For those who answered that their level of concern about privacy and security of the patient portal was high (ie, a score higher than 4 out of 7), they were additionally asked why they were concerned about privacy and security [22].

### Data Analysis

Descriptive analysis was performed using SPSS (version 28; IBM Corp) for each demographic and descriptive variable including mean, SD, frequency, and percentage.

A total of 1589 qualitative comments were collected on 4 open-ended questions: beneficial aspects of the patient portal (734 comments); difficulties in using the patient portal (179 comments); trust in privacy and security of the patient portal (554 comments); and concerns about privacy and security of the patient portal (122 comments). A combination of inductive coding and content analysis was conducted to elicit the main themes from the qualitative comments [23,24]. A set of coding rules were defined prior to the initial coding. The coding unit was a sentence, and the context unit was a question for each topic. The qualitative comments were coded into mutually distinct themes, and the frequency of coding units was calculated for each theme. If multiple sentences in a single comment referred to the same concept, the sentences were coded once as one unit. When a sentence included more than 1 concept, the sentence was coded multiple times for the applicable themes.

Using an Excel (Microsoft Corp) spreadsheet, 2 coders individually performed the initial coding following the same coding rules. The coders were nurse researchers with doctorate degrees who had conducted and published qualitative studies several times. Despite the use of predetermined coding rules to define the context unit and the coding unit, there was no overarching predetermined coding framework. Each coder independently derived mutually exclusive themes that emerged from the qualitative data. The coders compared their thematic results and discussed any coding discrepancies until a consensus was reached. As the coding progressed, the derived themes and unit frequencies were reviewed and refined through iterative discussions between the coders. A total of 22 themes on the 4 topics were finalized: beneficial aspects of the patient portal (6 themes); difficulties in using the patient portal (7 themes); trust in privacy and security of the patient portal (5 themes); and concerns about privacy and security of the patient portal (4 themes). Reliability related to the interpretation of word meanings was ensured by setting clear coding rules and following an iterative approach throughout the analysis. Semantic validity was achieved by assessing the correspondence between the categorization of the coding units and the question topics.

### Ethics Approval

Patients invited to the survey were able to decide to participate voluntarily. The survey research was approved by the institutional review board of University of Maryland, Baltimore (HP-00084885).

## Results

### Demographic and Descriptive Characteristics

Table 1 represents the demographic and descriptive characteristics of our sample (N=734). The mean age of the participants was 53.1 (SD 15.34, range 18-92) years. The majority of them were female (67.6%), White (68.5%), non-Hispanic (97.4%), and had some college or higher degree (83.3%). About two-thirds of them were married or living with a partner (62.9%), employed either full-time or part-time (57.7%), and had a monthly income of US $3000 or higher (60.2%). The majority of them reported having at least 1 chronic disease (86.5%); high blood pressure (46.8%) and high cholesterol (37.4%) were the 2 most reported chronic diseases. On average, the participants used the internet 24.9 (SD 20.78) hours per week. They showed relatively higher mean scores of eHealth literacy (mean 31.2, SD 5.51) and perceived usability of the patient portal (mean 36.6, SD 6.00) compared to previous studies with older adult web-based users [25,26]. Slightly less than half of them (47.3%) used the patient portal monthly or more frequently during the past 12 months. On a scale of 1 to 7, the mean score for a single item measuring concerns about privacy and security of the patient portal was 2.7 (SD 1.81); 18.7% selected a score value 5, 6, or 7, indicating “worried.”

**Table 1:** Demographics and descriptive statistics (N=734).

Variables		Values
**Age (years)**		
	Mean (SD)	53.1 (15.34)
	range	18.0-92.0
**Sex, n (%)**		
	Male	209 (32.4)
	Female	436 (67.6)
**Race, n (%)**		
	White	442 (68.5)
	African American	151 (23.4)
	Others^a^	52 (8.1)
**Ethnicity, n (%)**		
	Hispanic	17 (2.6)
	Non-Hispanic	628 (97.4)
**Marital status, n (%)**		
	Married or living with a partner	406 (62.9)
	Not married^b^	239 (37.1)
**Education, n (%)**		
	High school diploma or less	108 (16.8)
	Some college or college degree	338 (52.4)
	Graduate degree	199 (30.9)
**Monthly income (US $)**		
	<3000	241 (39.8)
	3000-4999	168 (27.7)
	≥5000	197 (32.5)
**Employment, n (%)**		
	Employed full-time or part-time	372 (57.7)
	Not employed^c^	273 (42.3)
**Having chronic disease, n (%)**		
	Yes	558 (86.5)
	No	87 (13.5)
**Number of chronic diseases**		
	Mean (SD)	2.5 (1.78)
	Range	0.0-11.0
**Chronic disease (yes), n (%)**		
	High blood pressure	302 (46.8)
	High cholesterol	241 (37.4)
	Arthritis	221 (34.3)
	Depression	184 (28.5)
	Diabetes	144 (22.3)
	Cancer	117 (18.1)
	Kidney problems	84 (13)
	Heart problems	79 (12.2)
	Osteoporosis	63 (9.8)
**Internet usage hours per week**		
	Mean (SD)	24.9 (20.78)
	Range	1.0-105.0
**eHealth literacy**		
	Mean (SD)	31.2 (5.51)
	range	8.0-40.0
**Patient portal use in the past 12 months, n (%)**		
	1 to 9 times	362 (52.7)
	About monthly	128 (18.6)
	More than monthly	197 (28.7)
**Perceived usability of the patient portal**		
	Mean (SD)	36.6 (6.00)
	Range	6.0-42.0
**Concerns about privacy and security**		
	Mean (SD)	2.7 (1.81)
	Range	1.0-7.0

^a^Others: American Indian/Alaska Native, Asian, Native Hawaiian or Other Pacific Islander, or more than 1 race.

^b^Not married: divorced, widowed, separated, and single.

^c^Not employed: retired, never worked, disabled, full-time student, homemaker, or self-employed.

### Beneficial Aspects of the Patient Portal

A total of 734 comments were entered into the analysis of beneficial aspects of the patient portal. Table 2 summarizes the themes with a coded comment frequency greater than 5% of the total comment frequency. The most frequently mentioned aspect was communicating with health care teams using the eMessaging function (n=279, 38%). They favored being able to communicate quickly and directly with their care providers on nonurgent matters without having to make an appointment or a phone call:

The ease of contacting my doctors. It eliminates the middle man and waiting that someone relays your question. I just send my doctor an email, and she responds right away.

ID 235

**Table 2:** Beneficial aspects and difficulties in using the patient portal.

Themes			Coded comments, n (%)^a^
**Topic: Beneficial aspects of your patient portal (n=734)**			
	Communicating with health care teams using eMessaging	279 (38)	
	Viewing test results and visit summaries	275 (37.5)	
	Managing appointments and receiving reminders	103 (14)	
	Easy access to personal health information	85 (12)	
	Getting prescription refills and reviewing medications	63 (9)	
	Ease of use and convenience	45 (6)	
**Topic: Difficulties in using your patient portal (n=179)**			
	Difficulty communicating with health care teams using eMessaging (eg, not getting responses timely and character limit)	45 (25)	
	Difficulty in the log-in process	31 (17)	
	Difficulty using patient portal functions (eg, appointment set up and medication refill)	24 (13)	
	Difficulty finding test results and other information	22 (12)	
	Information or list of health care providers not updated properly	15 (8)	
	Usability issues (eg, unclear display of information and difficult navigations)	14 (8)	
	Issues with patient portal system	10 (6)	

^a^Themes with a coded comment frequency greater than 5% of the total comment frequency are included.

Another aspect that was most mentioned was about viewing test results and visit summaries (n=275, 37.5%). The participants also favored the aspect of managing appointments and receiving reminders (n=103, 14%). They stated that these features help them keep track of their upcoming schedules and health status:

I like the ability to see my after-visit summaries and results, without having to call the provider’s office to try and track stuff down.

ID 719

What I like most is that it’s very convenient when scheduling appointments! And I love the fact that my test results are posted as soon as they come back!

ID 305

Eighty-five (12%) comments indicated easy access to personal health information as an advantage, like the following comment:

Availability 24 hours, ease of seeing information without need to bother office staff, able to print info to take with me to other doctors.

ID 373

Other featured beneficial aspects included getting prescription refills and reviewing medications (n=63, 9%) and ease of use and convenience (n=45, 6%).

### Difficulties in Using the Patient Portal

Of 187 participants who answered that they experienced difficulties when using the patient portal, 179 of them specified the difficulties they had (Table 2). Difficulties in communicating with health care teams via eMessaging were mentioned the most (n=45, 25%), including not getting timely responses and not being able to send messages to providers they want:

Some physicians and health providers don’t respond to patient message on the patient portal even don’t read the message and needs to call them and it waste a lot of time.

ID 848

Some providers do not use the email option and therefore makes it a bit more difficult to communicate with provider.

ID 429

Difficulties related to the eMessaging function itself, such as character limit or file attachment, were also mentioned.

The next most frequently mentioned was difficulty in the log-in process (n=31, 17%). They stated difficulties such as entering passwords multiple times or taking a long time to obtain a verification code:

At times I entered my password incorrectly and was contacted by the IT person and I was asked a lot of questions. Had to change my password.

ID 388

It takes 5 minutes to receive text for 3rd party verification to access the site, so by the time I get it, I’ve moved on to other things. I don’t have the time to sit around and wait to be able to enter a password.

ID 84

Twenty-four (13%) comments were about difficulties in using the patient portal functions to set up appointments and refill medications:

Scheduling appointments has been a challenge and the available listed is different than what they say on the phone.

ID 575

I tried to refill a prescription and the next day I couldn’t find any information as to my request being fulfilled. Had to call the doctor’s office to get refill completed.

ID 889

Another notable difficulty was finding test results and other information in the portal (n=22, 12%):

Tells me I have test results then there is no report.

ID 806

Looking through a lot of data to find out what I needed.

ID 1049

About 8% of the comments mentioned that information or list of health care providers is not updated properly (n=15, 8%). Another 8% were about usability issues (n=14, 8%), including unclear display of information and difficulty navigating the functions. Issues with the patient portal system were also cited as difficulty (n=10, 6%), for example:

The patient portal is always saying that it is deactivated. Then I must call to talk to a tech to help me get back on.

ID 919

### Trust in Privacy and Security of the Patient Portal

As shown in Table 3, a total of 554 participants submitted comments on what made them feel their information on the patient portal would be kept safe and private. The most mentioned comments were about trust in the health care system and network security (n=181, 33%). This includes trust in secure encrypted browsers (eg, assured by URL and Secure Sockets Layer lock icon) and faith in the health care providers and EHR or patient portal software company:

It is a hospital and doctor related site. You trust them and thus trust what they are asking us to use.

ID 585

The program is a world class EHR. I have few concerns about security with this system.

ID 782

**Table 3:** Trust and concerns about privacy and security of the patient portal.

Themes			Coded Comments, n (%)^a^
**Topic: Trust in privacy and security of your patient portal (n=554)**			
	Trust in health care system and network security	181 (33)	
	Protected registration and log-in process	173 (31)	
	Never been concerned or thought about privacy and security	107 (19)	
	Do not think that patient portal is fully safe and private but hope it is	57 (10)	
	HIPAA^b^ regulations, health care system’s privacy policies	40 (7)	
**Topic: Concerns about privacy and security of your patient portal (n=122)**			
	Risk of data breaches (eg, hacking attacks and identity theft)	74 (61)	
	Personal information could be used against me by third parties	17 (14)	
	Distrust of the internet/computer/patient portal system	16 (13)	
	Risk of others accessing my personal information	14 (11.5)	

^a^Themes with a coded comment frequency greater than 5% of the total comment frequency are included.

^b^HIPAA: Health Insurance Portability and Accountability Act.

Comments on security maintained by protected registration and log-in process were also frequently mentioned (n=173, 31%). The participants stated that personal verification is needed to sign up for the patient portal account, and personally owned information such as passwords or fingerprints is required to log-in. In particular, they appraised that the 2-factor authentication process strengthens the log-in security:

I needed a password or fingerprint to access my file.

ID 539

Having a security code that hopefully only the patient would be able to access.

ID 377

It has two layers of security features in order to login.

ID 241

About one-fifth of the comments mentioned that they had never been concerned or thought about the privacy and security of the patient portal (n=107, 19%). Many of them recognized that digital security is a web-wide concern, which cannot be completely guaranteed, for example:

It didn’t concern me, because I knew the information was already online, or on computers capable of going online. Me having access to it doesn’t increase the risk of it being stolen unless I personally make a mistake.

ID 707

Similarly, some participants commented that they do not think the patient portal is fully safe and private, but they hope it is (n=57, 10%):

In this age and time nothing is 100% safe, but I am hoping they are using the right safeguards to protect my information.

ID 318

Forty (7%) comments stated that federal statutes such as the HIPAA (Health Insurance Portability and Accountability Act) regulations and the health care system’s privacy policies established under applicable statutes ensure privacy and security:

Bound by HIPAA regulations, so I considered the process to be secure.

ID 361

### Concerns About Privacy and Security of the Patient Portal

Of 126 participants who had a high level of concerns about privacy and security of the patient portal, 122 commented on reasons for their concerns (Table 3). The majority of the comments were related to the risk of data breaches (n=74, 61%). The participants expressed their concerns about hacking attacks and identity theft occurring in health care institutions and private companies:

Because of past breaches of health information at the health care system, as well as breaches with personal credit cards, consumer credit reporting agencies, etc.

ID 822

It’s private information and the demographic data maintained could easily be used to steal an identity.

ID 322

Similar to concerns about data breaches, 17 (14%) comments particularly mentioned that their personal information could be used against them by third parties. They shared their experiences of personal information being compromised by insurance companies or other types of business:

Hackers, insurance using it against me later etc.

ID 228

My data has been compromised multiple times. I have had at least four fraudulent credit cards taken out in my name. I feel my health info is just as vulnerable.

ID 756

Distrust of the internet/computer/patient portal system was mentioned in 16 comments (13%), like stating:

I’m always worried about Internet/information security. It’s one of the biggest issues of our day.

ID 138

The risk of others accessing my personal information on the internet was mentioned in 14 (11.5%) comments:

I’m always worried about my personal health information being in multiple places (creates extra opportunities for people to get ahold of it who shouldn’t have access).

ID 116

## Discussion

### Principal Findings

Our findings provide an empirical understanding of the lived experience of using a patient portal in adult patient users across care settings, focusing on the beneficial aspects and difficulties in using the patient portal, and trust and concerns about privacy and security. The majority of the participants perceived the patient portal functions as beneficial for communicating with health care teams and monitoring health status and care activities. At the same time, about a quarter of them shared specific difficulties they experienced while using those functions. Although the level of concerns about privacy and security was generally low among the participants, they provided practical feedback that the software company and health care system personnel could refer to in order to implement the patient portal more secure and private.

The beneficial aspects and difficulties in using the patient portal found in our study were fairly consistent with what we found in previous studies [2,8,15,27]. Interestingly, some participants perceived a certain patient portal function as beneficial, while others found it difficult to use the same function. Communicating with health care teams using the eMessaging function was the most preferred feature among the participants, but it was also the most mentioned difficulty. This is probably because the eMessaging function is one of the most frequently used patient portal functions by patients [28,29]. Our participants favored direct communication with care providers for nonurgent matters. On the other hand, there were participants expressing difficulties in not getting timely responses or not being able to send a message to the care providers they want. This finding emphasizes the importance of care providers’ involvement in using a patient portal as patients’ experiences of using the eMessaging function may largely depend on care providers’ use of that function [2,16].

Viewing test results and visit summaries was another aspect that was most mentioned as beneficial in terms of tracking health status, but about 12% of those who had difficulties in using the patient portal experienced difficulty finding such information in the portal. Different perceptions also coexisted for other patient portal functions such as refilling medications and scheduling appointments; there were participants who found these functions convenient and useful, while others felt that those functions need further improvement. These differences in perceptions could be attributed to each participant’s eHealth literacy, proficiency with HIT, and usability of the patient portal, which have been reported as factors associated with the adoption of patient portals [8-11]. Indeed, those who had difficulties in using the patient portal had lower mean scores of eHealth literacy (*P*>.39) and perceived usability (*P*<.001) than those who did not. Periodic evaluation of the usability by end users with different levels of eHealth literacy would help make the patient portal more user friendly. Timely updates of accurate information on patient portals by health care teams may also help mitigate difficulties in viewing medical records and using medication refill and appointment functions.

The log-in process was the second most frequently mentioned difficulty. Since only 4.2% (n=31) of our sample mentioned this difficulty, we may consider that the configuration of the patient portal satisfies overall ease of access, although there is still room for improvement. Similar to previous studies [8,30], there were participants pointing out the inconvenience of entering usernames and passwords every time they log in to the patient portal and having to contact the service desk when the account is locked after entering them incorrectly. They particularly commented that it often takes a long time to receive a separate code via email or text for 2-factor authentication at log-in. Ironically, this log-in process was recognized by about a quarter of our participants as a key factor in making them trust that privacy and security are maintained on the patient portal. Health care institutions and IT developers should focus on balancing the convenience of logging in desired by users with the maintenance of security standards on the patient portal system.

Trust in the health care system and network security was most mentioned as what made the participants feel their information on the patient portal would be kept private and safe. Along with secure encrypted browsers, their faith in the health care institution and providers and the software company led to their trust in privacy and security of the patient portal. This aligns with the literature that a high level of trust in health care providers is an antecedent of fewer concerns about privacy and that providers' encouragements positively influence each individual's acceptance and use of patient portals [31-33]. Of the 554 submitted comments on this topic, 7.2% of them mentioned HIPAA, which is a federal law enacted to protect individuals’ sensitive health information [34] and the health care system’s privacy regulations. Further research is recommended to assess the impact of raising knowledge and awareness of personal health information safeguards on patients’ trust in privacy and security of patient portals [33].

Of the 122 participants who commented on concerns about the privacy and security of the patient portal, about two-thirds of them mentioned the risk of data breaches. They were aware of hacking attacks reported by the media and shared their direct and indirect experiences of identity theft. They were particularly concerned about the potential risk of personal information being leaked and used by private entities such as insurance and credit card companies. Indeed, data breaches have become a serious threat in the health care sector. The number has increased steadily over the past decade; 4419 health care data breaches of 500 or more records have been reported in the United States between 2009 and 2021 [35]. There was another perspective that distrust of the internet system leads to concerns about privacy and security. However, about 22% of our participants did not think much about this matter, stating that nothing is completely safe on the internet. This rather fatalistic view seems similar to the fact that many people are aware of the potential risk of electronic financial transactions, yet accept the risk and use digital banking [32]. Health care institutions and software companies implementing patient portals should continuously monitor the privacy and security safeguards of patient portals and provide relevant information assurance when necessary.

### Limitations

This study has several limitations. The survey recruited adult patients from a variety of 20 care settings from hospitals and clinics in a single health care system located in the mid-Atlantic region of the United States. The findings may not be generalizable since our sample and the patient portal (MyChart) included in this study cannot represent all patient portal users and programs. In addition, we only included those who had used the patient portal at least twice in the past 12 months prior to the survey; thus, infrequent users’ or nonusers’ perceptions of the patient portal were not reflected in this study. The relatively low survey response rate (7.5%) is another limitation that may affect the external validity of the study findings, although our response rate was similar to previous studies that used patient portal eMessages for participant recruitment [36,37].

### Conclusions

This study investigated the lived experience of using a patient portal in adult patients recruited from multiple care settings in a large integrated health care system, focusing on their perceived benefits and difficulties in using the patient portal along with their perceptions on privacy and security. The findings showed that most participants recognized the convenience, ease of use, and usefulness of the patient portal functions for communicating with health care teams and tracking care activities. About a quarter of the participants shared their difficulties in using the patient portal functions in terms of eMessaging communication, log-in process, and finding information in the portal. While the participants’ concerns about the privacy and security of the patient portal were generally low, they provided insightful comments that could help health care institutions and software companies implement patient portals to be more secure and private. Future studies may consider targeting other patient portal programs and patients with infrequent or nonuse of patient portals.

## Abbreviations

EHR: electronic health record
HIPAA: Health Insurance Portability and Accountability Act
HIT: health information technology
